# Effectiveness of a Short Mentalization Video Feedback Intervention Aimed at Adolescent and Young Mother–Infant Dyads: A Pilot Study

**DOI:** 10.3390/children13010044

**Published:** 2025-12-29

**Authors:** Elena Ierardi, Maria Elena Magrin, Alessandro Albizzati, Margherita Moioli, Renata Tambelli, Cristina Riva Crugnola

**Affiliations:** 1 Department of Psychology, University of Milano-Bicocca, 20126 Milan, Italy; 2 ASST Santi Paolo Carlo Hospital, 20142 Milan, Italy; 3 Department of Dynamic, Clinical and Health Psychology, University of Roma Sapienza, 00185 Rome, Italy

**Keywords:** adolescent and young mothers, mind-mindedness, disrupted communication, video feedback intervention, young motherhood risk factors

## Abstract

**Background/Objectives**: Early motherhood is a risk factor for the mother-child relationship during the perinatal period, implying that intrusive or withdrawn maternal behavior and possibly abusive behavior can have short- and long-term consequences for child development. **Methods**: This study aimed to evaluate the preliminary effectiveness of a short mentalization community video feedback intervention designed to improve maternal mentalization, maternal communication, and behavior quality of adolescent and young mothers. Twenty-six young and adolescent mother-infant dyads were recruited at an Italian outpatient hospital service for adolescent and young parents with a one-group pre-test/post-test design. The participants (*n* = 15) received four video feedback sessions focused on mentalization and affective communication. At 3 infant months, risk factors associated with young motherhood were assessed. At 3 infant months (pre-intervention) and 9 months (post-intervention), the quality of maternal communication and behavior has been evaluated with Atypical Maternal Behavior Instrument for Assessment and Classification (AMBIANCE), and maternal mind-mindedness was assessed. **Results**: At the end of the intervention, the results showed significant improvements in maternal communication quality and mind-mindedness, especially in decreasing disrupted maternal affective communication (*p* = 0.005) and non-attuned mind-mindedness comments (*p* = 0.005). **Conclusions**: This study highlighted good acceptance of the intervention in a public health context and the effectiveness of a short mentalization community video feedback intervention to improve maternal mind-mindedness and communication quality between young mothers and their infants. The preliminary findings of this pilot study could be useful for implementing similar interventions aimed at young parents in community contexts.

## 1. Introduction

Becoming a parent during adolescence and young adulthood is a complex experience that can cause intense distress and a significant impact on the parent-child relationship in the perinatal period [1,2]. Young parents find themselves facing potentially conflicting life changes: the transition to adulthood with the consolidation of their own identity and the transition to parenthood, involving the care of a newborn with intense physical and emotional needs [3].

During the perinatal period, young mothers may experience conflicting feelings: joy and enthusiasm for the newborn and, at the same time, concern over their new responsibility, with feelings of mistrust and low self-esteem [4]. They may also experience intense feelings of loneliness with respect to their peers and exclusion from the typical life of a teenager or a young adult. Furthermore, cognitive and neurophysiological development in young parents is still ongoing [5], implying less cognitive and decision-making competence in terms of assuming their parental role and less knowledge of their children’s developmental stages than adult parents [6]. The negative impact of early parenthood is also linked to multiple associated risk factors [7,8]. In fact, young mothers often have a low socioeconomic status and a low level of education. They are also more likely than adult mothers to be single parents, often having unstable relationships with the child’s father. Furthermore, they frequently do not enjoy sufficient social support from their families, which are themselves characterized by a history of early parenthood [9,10]. Many young mothers also experience physical and sexual abuse and physical and emotional neglect during childhood and early adolescence [11,12]. This means that they are highly likely to engage in inappropriate and potentially abusive behavior toward their newborns [13].

Moreover, adolescent mothers often have mental health issues during the perinatal period. Compared with adult mothers, they are 50% more likely to display high levels of depression [14,15,16] and anxious states [17]. A high level of posttraumatic stress disorder (PTSD) due to frequent traumatic experiences and a high probability of substance abuse have also been reported [18].

The highlighted psychological vulnerability has a significant effect on how young mothers relate to their babies from the first months of life [19] because pregnancy is often unwanted or unplanned [20]. For these reasons, the first encounter with the child can be problematic, causing feelings of inadequacy in the mothers. Young mothers often find it difficult to attune with their child and understand their needs [21], oscillating between hostile and intrusive behavior and detached behavior [22]. In the most problematic cases, this behavior can be associated with physical and verbal abuse [13]. They may also have difficulty comforting the infant and regulating his/her distress [3]. Young mothers also frequently show a poor ability for mentalization [23], both regarding reflective functioning, understood as the ability to recognize one’s own and others’ mental states [24], and to mind-mindedness, understood as the ability to think of the child as having a mind and to attribute mental states to him or her [25]. This is particularly relevant since parental mentalization was found to be correlated with both maternal responsiveness and secure infant attachment [26].

Owing to these unfavorable conditions, children of adolescent and young parents frequently develop adverse trajectories, establishing insecure and disorganized attachment [27] and showing delays in language and cognitive development [28,29]. Once they reach adolescence, they are more likely to become young parents themselves and to show antisocial behaviors [30,31].

Early motherhood, at the same time, has an impact on the life cycle of young mothers [32], hindering the achievement of a good level of education and employment and leading to poor relationships and socialization [33,34].

Despite the numerous risk factors associated with early motherhood, the literature has highlighted some protective factors that can facilitate the experience of parenthood and the relationship with the child for young parents. Among these, the following appear to be important: enjoying adequate social support from the family of origin, the partner and the group of peers and friends [4], being supported in completing one’s studies, and finally, having experienced closeness and warmth in childhood [1]. Another aspect that facilitates the experience of parenthood appears to be having access to primary care during pregnancy and the perinatal period which does not stigmatize young individuals [22,34].

The presence of these protective factors can make the experience of motherhood a turning point for young mothers, in relation to the definition of their identity, a process still in progress given their adolescent or young adult age, and to the reduction of their possible risky behaviors prior to birth. Recent research conducted using mixed quantitative and qualitative methods has highlighted how several of the teenage mothers interviewed experienced motherhood as something that significantly promoted their personal growth and maturation.

Given the risk associated with early motherhood and its impact on the relationship with the child, it seems important to implement interventions during the perinatal period aimed at facilitating the mother’s relationship with the child by promoting a secure attachment bond. Importantly, secure infant attachment is predictive of an infant’s positive socioemotional development and is considered a protective factor with respect to future psychopathological problems [35]. Similarly, maternal responsiveness and the absence of controlling or avoidant styles of interaction toward the child have a lasting effect on the child’s socioemotional development [36].

Several programs aimed at improving the relationship between adolescent and young mothers and their infants have been developed [37]. Different approaches have been taken, including home visiting programs [38,39], and clinical based programs, which are finalized to monitor parents’ and children’s well-being and to provide medical assistance and social support [40]. Among these, particularly relevant is the *Minding the Baby Program* (MTB) [41], a longitudinal mentalization-based intervention carried out in the perinatal period. Several of these programs have shown significant results for both young mothers and children. In particular, the MTB program achieved positive outcomes at the end of the intervention in terms of both the mentalization capacity of mothers and the security of children’s attachment [41].

However, few interventions tailored to the specific needs of young parents have been implemented in the community [42]. The intervention program we tested in a hospital outpatient service for adolescent and young parents is a short community intervention based exclusively on a video feedback technique. It is an intervention tailored to young mothers, as its main objectives are to increase the quality of the relationship with their children, and to prevent the emergence of disrupted communication characterized by affective errors, role/boundary confusion, fearful disorientation, and intrusive/negative and withdrawal interactions [43], which are typical of mothers in high-risk conditions and with adverse childhood experiences [44,45], and enhance their mentalization capacity. The capacity for mentalization and affective communication can in fact—as highlighted above—be weak in young mothers. The short duration of the intervention, which takes place during a crucial period for the formation of early parent-child relationships, also aims to reduce the frequent drop-out rates seen in longer longitudinal programs targeting adolescent high-risk parents [46].

## 2. Video Feedback Intervention

The video feedback technique aims to support parent-child relationships by improving parenting skills. The common goal of interventions based on this technique, beyond the different theoretical backgrounds, which mainly consists of attachment theory and social learning theory [47], is to promote, by adopting a third-person perspective, a greater understanding of parents’ emotional and mental states underlying their own and their children’s behaviors, promoting their sensitivity and awareness [48].

Owing to the use of the video, parents usually benefit greatly from the opportunity to observe themselves interacting with their child, as this allows them to express and become aware of their mental and emotional states in relation to themselves and their baby, and thus, activate specific resources and strengths [49,50]. Over the years, the literature has shown that video feedback is effective in promoting maternal responsiveness and a child’s secure attachment bond to the mother [47,51].

However, few studies [52] have evaluated whether the use of video feedback can increase a parent’s mentalization capacity, understood as both parental reflective functioning and mind-mindedness. Among these, the MTB home visiting intervention, aimed at mothers with adverse experiences and psychosocial risk, which is based on video feedback integrated with other techniques, has been shown to promote maternal reflective functioning and responsiveness [41,53].

Similarly, only one study, to our knowledge, has verified that video feedback intervention can modify the disrupted quality of maternal communication toward the child observed in free interaction before and after the end of the intervention [54]. In Slade’s [41] study on the efficacy of the *Minding the Baby* program, the interactions of the recruited mothers were evaluated with the AMBIANCE system only at 4 months and not at the end of the intervention. Tereno [45] and Yarger [55] evaluated the effectiveness of a home visiting intervention at the end of the intervention by analyzing disrupted maternal communication in the *Strange Situation* and compared the intervention group with a control group. Importantly, in general, most studies on the effectiveness of video feedback are based on maternal sensitivity as an outcome, even in at-risk samples, without considering disrupted aspects of maternal communication.

In recent years, brief video feedback interventions (between three and six sessions) have been conducted with mothers in low-risk conditions [56] and high-risk conditions, such as postpartum depression [57] and traumatic experiences [58]. Other studies have shown that short video feedback interventions are more effective at improving parenting skills than longer-term programs [46,59]. Notably, short-term interventions aimed at parents during their children’s infancy have generally been found to be more effective than long-term interventions in increasing maternal sensitivity [60,61,62].

## 3. Methods

### 3.1. Aims

The preliminary exploratory object of our study was to assess the risk factors present in young mothers—for whom the video feedback intervention has been proposed—in terms of mind-mindedness, psychological distress, attachment styles, social support, and affective communication.

The principal aim of the study, on the basis of what has been considered thus far, was to show preliminary results on the effectiveness of the four-session mentalization video feedback intervention used as a stand-alone technique for improving the maternal–infant interaction quality and maternal mind-mindedness of adolescent and young mothers attending an outpatient hospital service for young parents and their infants. A further objective of the study was to evaluate the acceptability of the intervention with this group of mothers at risk.

Specifically, we hypothesized that the intervention would be effective in improving the quality of maternal communication, reducing disrupted communication as assessed by the AMBIANCE system. According to Lyons-Ruth [43], disrupted parental communication is characterized by various aspects, such as communication errors, role/boundary confusion, fearful/disoriented behavior, intrusive/negative interactions, and withdrawn interactions. Disrupted parental communication has been shown to be predictive of disorganized attachment of the child to the parent [43,55] and therefore to be a risk factor for the child’s socioemotional development and the emergence of psychopathological problems [63]. At an exploratory level, we consider which of the various aspects of disrupted communication mentioned above may decrease at the end of the intervention.

We also hypothesize that the intervention is effective in improving the quality of maternal mind-mindedness, contributing to an increase in mind-related attuned comments and a decrease in non-attuned comments. By mind-mindedness, we mean, following Meins and Fernyhough [25], the parent’s ability to attribute internal states to the child by commenting on their activity and communications through verbal mind-related comments. Such comments may be attuned to the child’s activity or, conversely, not attuned. A high frequency of maternal mind-related attuned comments has been found to be correlated with a child’s secure attachment to the mother, whereas a high frequency of mind-related non-attuned comments has been found to be predictive of disorganized attachment [64].

### 3.2. Short Mentalization Video Feedback Intervention

The short mentalization video feedback intervention we have tested was developed based on the literature that emphasizes the importance of parental mentalization [24] in building a secure attachment bond between parents and children and, consequently, on the usefulness of programs focused on mentalization in the perinatal period [65,66]. The intervention is tailored to the specific characteristics of young mothers, implying poor sensitivity, disrupted communication and a low ability to read and attribute emotional states and intentions to the child with the aim of improving parental mentalization. The six-month intervention can be considered short compared with most interventions aimed at mothers at risk, which often use a longitudinal approach.

The use of video feedback has proven to be a useful and cost-effective tool for improving maternal sensitivity and mentalization [52], even with a low number of sessions. The shortness and outpatient nature of our intervention also aims to address the difficulties young mothers face in accessing public hospital services for parents in the perinatal period [34,41].

The main goals of the intervention are therefore to enhance mothers’ mind-mindedness and to improve the quality of their communication with their children, reducing communication errors and intrusive or withdrawn behaviors, role boundary confusion, and fearful or frightening behaviors.

The intervention is carried out in an outpatient service of an Italian hospital aimed at adolescent and young mothers. Mothers were contacted after giving birth and offered free participation to take part in a program to support their relationship with their child. The short video feedback intervention lasted 6 months, from the baby’s 3rd month until the 9th month.

The starting point of the intervention was collocated 3 months after childbirth, considering the physiological, psychological, and relational adjustment of mother-infant dyads, which characterizes the early postpartum period.

The young mothers received four video feedback sessions conducted at 6-week intervals. The outcome data were collected at pre-test at 3 months (prior to the intervention) and post-test (post-intervention at 9 months).

The psychologist’s approach to discussing the video with young mothers is characterized by simple and informal language, with nonjudgmental questions aimed at welcoming any type of response, starting from a behavioral/concrete level to explore emotional and mental levels as well.

The core aspects of the intervention are as follows: 5 min of free play mother-infant interactions are videotaped; psychologists edit the recording and select the interactive episodes exemplifying the infant’s’ communication as well as maternal responses to them; assisted a view of the recordings is carried out in each session with the young mothers by the psychologist (see Table 1).

During the four sessions, the psychologist discussed the video with the mother, adopting a “reflective stance” to enhance her mentalization [49]. Young mothers are asked to observe their own and their child’s behavior and mutual responses as well as the emotions they feel. The aims of the discussion are to help the mother understand her own mental state and her child’s mental state (feelings, intentions, and needs) underlying her interaction and to enhance her responsiveness and the quality of her affective communication (see Table 2). In the discussion, following the literature on the use of video feedback [67], the starting point is positive moments of interaction between mother and child, moving on later to explore negative moments.

Finally, psychologists were specifically trained on issues related to young parenting and video feedback interventions. Intervision sessions were also planned every two weeks to monitor and improve the quality of the intervention but not to implement fidelity.

### 3.3. Design

A one-group pre-test and post-test design was used to evaluate the effectiveness of the short mentalization video feedback intervention aimed at young mothers and their infants [68].

### 3.4. Participants

The research took place at “Servizio di Accompagnamento alla genitorialità in adolescenza” [Accompanying Parenting in Adolescence Service] aimed at parents under 22 years of age at the ASST Santi Paolo and Carlo Hospital in Milan, Italy. This is a free service offered by the Infant Neuropsychiatric Unit of the Hospital with the aim of supporting young parents and their infants during the first two years of their child’s life. The participants were 26 young and adolescent mother-infant dyads; the mothers were between 15 and 21 years old (M = 18.69, SD = 2.25).

The inclusion criteria for participation in the study included the following: ability to speak and understand the Italian language; age range between 14 and 21 years; uneventful delivery; birth at full term with no medical complications and being physically healthy. The exclusion criteria included prematurity and twin birth.

The infants (male infants = 14) were all born full term, without organic pathologies. Mothers were mainly Italian (57%) and the remaining mothers were European and Latin American and were aware of the Italian language and were integrated into the Italian cultural context.

### 3.5. Procedure

We contacted and invited 37 adolescent and young mothers with their infants to participate in the intervention. Mothers interested in participating in the research signed the informed consent form at the initial meeting. After an initial meeting, 30% of mothers did not return due to a lack of interest in the intervention. In addition, 19% did not participate after two subsequent meetings with 2 sessions of video feedback. The assessment carried out at the end of the intervention was completed by 57% (15/26) of the mothers who demonstrated good adherence to the intervention.

The characteristics of those who completed the intervention compared with those who did not differ in terms of maternal age, child gender, presence of a partner, level of education, socioeconomic status, nationality, and whether the pregnancy was desired.

In 23% of the cases, they had yet to complete the intervention at the time of our data collection, having held only a few meetings thus far.

Five minutes of video recording during free play interaction were well accepted by the mothers, who provided good material for the video feedback intervention. The technological requirements for implementing the intervention are easily accessible to primary professionals; what is required is a personal computer, and a video camera.

### 3.6. Measures

#### 3.6.1. Sociodemographic Profile

To evaluate sociodemographic characteristics, an ad hoc anamnestic form was created to analyze: socioeconomic level, level of education, with whom they live or if they live in a residential community, desired/unwanted pregnancy, presence/absence of the partner, history of parenthood at a young age, unemployment, and presence of child social services.

#### 3.6.2. Edinburgh Postnatal Depression Scale

The Edinburgh Postnatal Depression Scale (EPDS) [69] is a self-report questionnaire that assesses the presence of depressive symptoms. In this study, we used the Italian version and the clinical cutoff (between 9 and 12 medium, 13 or more high), as indicated by the Italian validation.

#### 3.6.3. Generalized Anxiety Disorder Scale

The Generalized Anxiety Disorder Scale (GAD-7) [70] is a self-report questionnaire that assesses generalized anxiety disorder symptoms. In this study, we used the Italian version.

#### 3.6.4. Multidimensional Scale of Perceived Social Support

The Multidimensional Scale of Perceived Social Support (MSPSS) [71] is a self-report instrument that measures perceived social support from family, friends, and a particularly significant person. The scale comprises a total of 12 items, with 4 items for each subscale M. The total scores divided by 12 items put the subject into 3 groups based on their scores (trichotomize) and designated the lowest group as low perceived support, the middle group as medium perceived support, and the high group as high perceived support. In this study, we used the Italian version.

#### 3.6.5. Relationship Questionnaire

The Relationship Questionnaire (RQ) [72] is a 4-item questionnaire designed to measure adult attachment style. The RQ extends the original attachment three-category measure [73] by rewarding the descriptions of each of the attachment styles and by adding a fourth style dismissing-avoidant.

The RQ has four measurable categories of attachment style: secure, fearful, preoccupied, and dismissing. There are two parts, RQ1 and RQ2. In the first part, RQ1, participants were asked to select a paragraph-long description that best described them without providing a numerical rating. In the second part, RQ2, participants are asked to rate their agreement with each prototype on a 7-point scale. The highest of the four attachment prototype ratings is then used to classify participants into an attachment category. In this study, we used the Italian version.

#### 3.6.6. Mind-Mindedness

Maternal mind-mindedness was assessed from a video-taped 5-min free-play session. Mothers’ speech during the sessions was transcribed verbatim; the comments were divided into comments not referring to the infant’s mind or emotion (not mind-related comments) and comments that included an internal-state term referring to the infant’s mind or emotion (mind-related comments). Mind-related comments included references to wishes and desires, mental states, mental processes, emotions, and comments where the mother ‘‘put words into her infant’s mouth’’. Attuned mind-related comments were judged to be consistent with the infant’s observed behavior, comments linked the infant’s current activity with similar events in the past or future, comments served to clarify how to proceed if there was a lull in the interaction, or the mother voiced what the infant might say if he/she could speak [25]. (e.g., “Would you like to take the blue stuffed animal that you like so much?” “Aren’t you interested in the book at all?” “Hi, Mom, I want to play with you!”).

Non-attuned mind-related comments should be coded when the researcher disagrees with the caregiver’s reading of the infant’s current internal state, the comment refers to a past or future event that is unrelated to the infant’s current activity, the caregiver asks what the infant wants to do or suggests that the infant wants to become involved in a new activity when the infant is already actively engaged in playing with or attending to something else, the caregiver seems to be attributing internal states (epistemic states, emotions or desires) that are not implied by the infant’s behavior and that appear to be projections of the adult’s own internal states onto the child, and the referent of the caregiver’s comment is not clear [25]. (e.g., “You’re bored with that one” referring to a toy with which the infant is still actively playing “Are you tired?” after the infant has shown no overt signs of tiredness).

The mind-mindedness score was the number of mental descriptors expressed as a proportion of the total number of descriptors used to control differences in maternal verbosity. Higher scores indicate greater mind-mindedness. To assess interrater reliability, a random selection of transcripts (20%) was scored by two raters who were blinded to the other variables considered. Interrater reliability was as follows: K = 0.90 for mind-related comments and K = 0.92 for attuned mind-related comments.

#### 3.6.7. AMBIANCE

The Atypical Maternal Behavior Instrument for Assessment and Classification (AMBIANCE) [74] is an observational coding system designed to assess the quality of affective communication between mothers and infants and specific disrupted caregiving behaviors. Videotaped face-to-face interactions are coded on five dimensions of disrupted behaviors: affective communication errors, contradictory communications or failures to respond to clear infant cues, especially cues for comfort; role/boundary confusion, behavior that prioritizes the parent’s needs over the infant’s needs, or more rarely, sexualized behaviors toward the infant; fearful/disorientation, behavior that appears frightened, dissociated, or affectively odd; intrusiveness/negativity, behavior that is frightening or threatening, that communicates a hostile attitude toward the infant, or that interferes with the infant’s ongoing directions; and withdrawal, behaviors that communicate reluctance to interact fully with the infant [43]. The frequency counts for each dimension were then compared and matched to existing qualitative descriptions for each dimension. These qualitative descriptions exist on a 7-point scale ranging from 1 = not disrupted to 7 = disrupted caregiving with few ameliorating behaviors. Next, the ratings for each dimension are considered to obtain an overall score for disrupted caregiving, ranging from 1 = “undisrupted, warm and sensitive communication” to 7 = “very disrupted caregiving.”. The ICC was 0.86 for the disruption scale score.

## 4. Data Analysis

The SPSS Statistic 29 package was used for all analyses. Descriptive statistics were calculated with respect to demographic characteristics and risk factors at pre intervention phase. To evaluate the effectiveness of the intervention, owing to the small sample size, the Wilcoxon test was used to identify the differences between pre and post intervention with respect to mind-mindedness and AMBIANCE.

## 5. Results

### 5.1. Young Mothers’ Risk Factors

SES was low in all cases, and 70% of the mothers left school at the age of 16. Unwanted pregnancies resulted in 65% of the mothers. Moreover, 90% of the adolescent mothers had mothers who also had early pregnancies. Nineteen percent of them lived in mother-child residential care facilities, 38% lived with their partner, and 19% lived with their parents. In 38% of the mother–infant dyads, the child’s social services were present.

Regarding psychological distress, a total of 47% of the mothers had symptoms of postpartum depression, 35% of whom were in the subclinical range and 7.6% of whom were in the clinical range. With respect to anxiety, 38% of the mothers had mild anxiety, 7% had moderate level of anxiety, and 15% had severe anxiety.

Regarding social support, 36% of the mothers perceived medium social support, and 54% perceived high social support.

With respect to maternal attachment style, mothers had a prevalence (55%) of fearful/avoidant attachment style. Ten percent of the remaining participants had a secure attachment style, 27% had an Anxious/Ambivalent attachment style, and 8% had an Avoidant attachment style.

About the affective communication quality, the AMBIANCE Total score indicated that 50% of the mothers experienced a disrupted level of communication.

Attuned mind-related comments of the young mothers were less frequent (M = 0.02; SD = 0.02), and the non-attuned mind-related comments were more frequent (M = 0.10; SD = 0.28) when compared at a descriptive level with those of not-at-risk mothers in the Meins study [75].

### 5.2. Intervention Effectiveness

To test the effectiveness of the intervention on maternal affective communication and mind-mindedness, the Wilcoxon test was used, which revealed significant differences (see Table 3).

Regarding the AMBIANCE dimensions, a significant decrease in the total score was found between the pre-intervention and post-intervention phases, confirming our hypothesis. In addition, there was a significant change in the role boundary/confusion dimension, with a decrease in the scores of this dimension between 3 and 9 months. Moreover, at a descriptive level, all AMBIANCE dimensions, except for affective communication errors, showed a reduction in scores. Regarding the categorical distribution, in the pre-intervention phase, 50% of the mothers had a score falling within the disrupted level of the communication category and 50% in the non-disrupted category; in post-intervention, only 33% fell within the disrupted level of the communication category and 67% in the non-disrupted category, with significant variation (Fisher’s exact test = 6.56; *p* = 0.026).

Regarding mind-mindedness, a significant decrease in non-attuned mind-related comments was found from pre-intervention to post-intervention phases, confirming the initial hypothesis. Moreover, although at a descriptive level, there was an increase in attuned mind-related comments after 4 sessions of intervention.

In summary, the study highlights a profile of the risk and vulnerability of young mothers assessed at three months of their child’s age, concerning both psychosocial aspects and psychological distress, as well as relational and mentalization skills.

Regarding the intervention, the results show a significant improvement in mothers’ mind-mindedness and communication skills after its completion, with a decrease in disrupted maternal affective communication and non-attuned mind-mindedness comments.

## 6. Discussion

This study highlights the high-risk profile of the young mothers involved in the study and the acceptability and effectiveness of a short mentalization video feedback intervention to increase young and adolescent mothers’ quality of mind-mindedness and affective communication.

The profile of the young mothers included in the study revealed that they were at high risk in terms of psychosocial factors, psychological distress, attachment patterns, mentalization abilities, and affective communication. These results expand the existing literature, that highlights how young motherhood is characterized by multiple interconnected risk factors [2,76], providing a multifaceted view of early motherhood and revealing unexplored aspects, including attachment styles, mind-mindedness, and disrupted affective communication.

In fact, at 3 months of infant age, before the intervention, the young mothers in our sample had significant social risk factors, such as low socioeconomic status and low educational level. The housing situation is also often problematic, so in 19% of cases, children’s social services were involved, and the dyads had been placed in a mother-child residential care facility. Furthermore, in more than half of the cases (65%), pregnancy was unwanted.

In terms of psychopathological vulnerability, almost half of the young mothers exceeded the cut-off for depressive symptoms, and almost 40% exceeded the cut-off for anxiety. These percentages are more than double the risk of postpartum depression and anxiety in normative samples [77]. As is well known, postpartum anxiety and depression are risk factors for the development of an inadequate parent–child relationship, a child’s insecure attachment, and the development of psychopathological symptoms [78].

Interestingly, young mothers mostly exhibit a fearful/avoidant attachment style characterized by a combination of anxiety and avoidance, which involves difficulty establishing intimate relationships and feeling comfortable with closeness and intimacy [72].

Maternal mind-mindedness was also low with frequent non-attuned mind-related comments, indicating difficulty in thinking about their children in terms of internal states, such as desires and intentions, as well as misunderstanding their infant’s states of mind. This finding is particularly significant given that a high frequency of non-mind-related comments is associated with disorganized attachment of the child to the parent [64]. This type of attachment is also a risk factor for a child’s development in terms of stress dysregulation, externalizing disorders [63,79] and antisocial and suicidal behavior in adolescents [36].

All these factors were linked to maternal difficulties in achieving an adequate level of affective communication in interactions with infants, as indicated by the fact that half of the mothers fell within the disrupted range of AMBIANCE regarding mothers’ level of communication at 3 months. Therefore, the difficulties faced by young mothers in parenting styles seem to be multilayered in terms of interaction, communication and mind-mindedness.

The only protective factor identified was mothers’ perception of social support, with the feeling that they had at least one figure among family members, partners, and friends from whom they felt supported. It could be hypothesized that young mothers were unable to attribute the true meaning to social support, perceiving it incorrectly. This hypothesis needs to be explored further with a larger sample size and the use of other social support assessment tools.

For the outcome of the intervention, although preliminary, the pilot study shows that a brief mentalization-oriented intervention conducted in a public health context was effective in reducing disruptive affective communication. Regarding affective communication, it is also important to note that there was not only a significant decrease in the total AMBIANCE score but also in the role/boundary confusion scale, which involves role reversing behavior on the part of mothers toward the child. This scale indicates behavior and communication that prioritizes the parent’s needs over those of the infant, e.g., asking for reassurance or affection when the infant is distressed [45]. The intervention focused on improving parents’ ability to reflect on their child’s emotional states, could therefore be effective in making young mothers more attentive to their infants’ needs than to their own needs, as role confusion has been reduced. In this respect, it will be important to verify in a larger group whether this data will be confirmed or whether the other scales of AMBIANCE will also change significantly. In terms of mentalization, the intervention seemed to help decrease non-attuned mind-related comments of the young mothers, thus reducing the number of comments that are not attuned to the infant’s mental and emotional states. This result appears to be particularly important given that, as highlighted above, non-attuned mind-related comments were found to be predictive of disorganized attachment. The attribution of internal states that do not correspond to the child’s activity by the parent can also be considered, from a psychodynamic point of view, a risk for the development of the child’s personality, since it misinterprets and disregards crucial aspects of the child’s activity and communication [80].

Both results appear significant given that as highlighted above, non-attuned mind-related comments and disrupted communication by mothers were found to be potential predictors of disorganized attachment in subsequent developmental stages [5,43,64].

At a descriptive level, there was also an increase in the number of attuned mind-related comments. Future research with a larger sample will shed more light on whether the intervention can also be effective in increasing young mothers’ ability to make more attuned mind-related comments.

Finally, it is important to emphasize that the decrease in non-attuned comments (e.g., commenting that the child likes a toy when he or she is rejecting it) and the decrease in disrupted communication, which implies a lack of understanding of the child’s needs, seem to indicate that the mothers who participated in the intervention have changed aspects of their parenting centered on misunderstanding the child’s behavior at different levels, both in terms of affective communication and mind-mindedness. In this context, Meins and colleagues highlighted how there is a parallel between non-attuned mind-related comments and some atypical parenting behaviors described by Lyons-Ruth, such as affective errors and intrusion and withdrawal behaviors, suggesting that a parent who misreads the infant’s mental and emotional states could confuse and potentially scare the child [81].

Finally, the intervention was well accepted by the mothers involved, with more than 50% attending all the sessions.

The pilot study has several strengths. First, the study highlights the possibility of implementing brief intervention focused mainly on mentalization in a public health context and aimed at a group of mothers at risk, such as adolescent mothers, who show significant changes in mothers’ affective communication and mind-mindedness. The possibility of implementing a short intervention based on an easy-to-use procedure is important given that prevention programs aimed at high-risk parents are often difficult to implement over long periods of time despite having a significant effect on the well-being of parents and children and their social context [45].

The brevity of the intervention also makes it more accessible to young mothers, who, for their age, are known to give up programs that require long-term attendance [82].

Second, this is one of the few studies that evaluate mind-mindedness as an outcome measure in a brief mentalization-focused intervention. At the same time, it is one of the few studies that evaluates disrupted communication with the AMBIANCE system through pre/post measures in a high-risk sample such as adolescent mothers, given that most studies on effectiveness, despite targeting high-risk participants, focus on maternal sensitivity as an outcome. Importantly, unlike this study, several studies in the literature are based on self-report questionnaires rather than direct observations of child–mother interactions.

The study also has several limitations. First, the sample size is limited, as this is a pilot study that requires replication in larger samples; the small sample size could have an effect on the acceptability and statistical power of the results. Increasing the sample size could also overcome the problem that this small sample size leads to underpowered statistical analyses. Moreover, the lack of a control group and the nonrandomization of the study, owing to the difficulty of recruiting high-risk and vulnerable participants such as adolescent and young mothers, limit statements about the effectiveness of the intervention. Another limitation is that the coding of the mother-infant interactions was not blind: the age of the children (3 or 9 months) was easily identifiable based on physical characteristics and skills that evolved at different stages of development. Moreover, since only half of the mothers were Italians, this could be a potential cultural difference in parenting communication or access to support. Finally, the improvement in the quality of mother–infant relationship at the end of the intervention could be due to other variables, such as the child’s developmental maturation and the implicit nonspecific support given to the mothers.

As a future direction of study, it would be useful to consider further risk and protective factors for adolescent and young mother–infant interactions, such as parenting stress, maternal history of adverse childhood experiences, and child temperament, to analyze how these variables could moderate the intervention. By using a larger number of participants, it will also be possible to investigate possible correlations between depression and anxiety in young mothers and the quality of their emotional communication with their children. Another relevant direction of research could be to analyze children’s outcomes post-intervention such as their interaction style and attachment pattern to the mother.

## 7. Conclusions and Implications

This study offers preliminary evidence that a short video feedback intervention focused on mentalization aimed at vulnerable populations and carried out in a public health context could be effective in helping to improve different aspects of adolescent and young mothers’ parenting. In fact, on the one hand, the intervention has contributed to improving maternal mind-mindedness, i.e., the ability to attribute internal mental states to the child, and on the other hand, to reducing maternal disrupted communication. The preliminary data of this pilot study may therefore be useful for implementing short video feedback interventions aimed at high-risk populations such as adolescent and young mothers in different community contexts. Future research should evaluate with a longitudinal approach whether short-term intervention could improve mothers’ mentalization and affective communication over time, particularly in the child’s second year, and could also affect children’s development. Moreover, further research is needed to confirm these results and explore their applicability to other at-risk populations.

## Figures and Tables

**Table 1:** Description of the Short Mentalization Video Feedback Intervention.

Aims	The operational aims of the four sessions are: -to improve the mother’s understanding of her child’s communications and feelings -to reflect on mother’s feelings and states of mind about herself and her child -to increase her capacity for mind-mindedness, consistently attributing emotional and mental states to her child -to increase maternal responsiveness.
Setting	Few days before each session with the mother a 5-min video recording of free play interactions between mother and child is carried out in a room equipped with a video recorder in which age-suitable toys are available.
Procedure	Four one-hour sessions are conducted by a psychologist with the mother every 6 weeks, viewing and commenting on 2 or 3 clips edited by the psychologist extracted from the previously recorded video.
Focus	The clips, lasting each 10–30 s, concern: -positive interactions involving moments of face-to-face interaction with eye contact, exchanges of vocalizations, etc., or play with objects in which the mother’s responsiveness and the child’s cooperation are evident -moments of face-to-face interaction with eye contact, exchange of vocalizations, etc., play or with objects in which disrupted maternal communication emerges—e.g., the mother proposes to play when the child cries or the mother remains silent when the child smiles, etc.—and an unresponsive or rejecting response from the child.
Method	During each session, the psychologist: -focuses both on positive interaction between mother and child and on moments of interactive exchanges that do not work between parent and infant -asks the mother for comments regarding her interaction with the child and child’s responses -attempts to improve mentalization skills by asking the mother how she feels during moments of positive and negative interaction, helping her to understand her own emotions and feelings in relationship with the child and those of the child. To improve not adequate interaction, the psychologist reflects with the mother on possible more useful alternative strategies for interacting and playing with the child. During the sessions the psychologist also focuses on any new adaptive ways for mother and child of interacting and being together that appeared during the intervention.

**Table 2:** A brief example of video commentary by a psychologist with a young mother during a session of the intervention aimed at enhancing her mind-mindedness.

Psychologist and a young mother watch a clip of the videorecording together. In the clip the mother uses a book in an intrusive manner, repeatedly bringing it excessively closes to the face of her 3-month-old daughter, Sara, who after a few seconds closes her eyes and cries. Psychologist: *Why is Sara crying?* Mother: *I don’t know.* Psychologist: *Try to think about it.* Mother: *I have no idea.* Psychologist: *Try to put yourself in Sara’s shoes and imagine someone putting an object on your face. How would you feel?* Mother: *Um, it would bother me.* Psychologist: *So, try to think about how Sara felt.* Mother: *I would be annoyed, it wouldn’t be pleasant, I would say, “Mummy, stop, I don’t like”*

**Table 3:** Differences in mind-mindedness and AMBIANCE pre- and post-intervention.

Intervention Group (N = 15)						
	3 Months	9 Months				
	M (sd)	M (sd)	Z	*p*	r	95% CI
**AMBIANCE**						
D1 Affective Communication errors	2.87 (1.72)	2.93 (1.10)	0.32	0.74		
D2 Role/boundary confusion	2.33 (1.83)	1.20 (0.56)	−2.21	0.027 *	0.57	−2.0, 0
D3 Fearful/Disoriented Behaviors	2.67 (1.58)	2.07 (1.22)	−1.06	0.28		
D4 Intrusiveness/Negativity	3.27 (1.58)	3.20 (1.32)	−0.16	0.86		
D5 Withdrawal	3.00 (2.00)	2.87 (1.80)	−0.49	0.61		
Total scores	4.60 (1.32)	3.80 (1.20)	−2.83	0.005 **	0.07	−1.0, −0.5
**Mind-Mindedness**						
Attuned mind-related comments	0.02 (0.02)	0.04 (0.04)	1.64	0.107		
Non-attuned mind-related comments	0.12 (0.24)	0.00 (0.00)	−2.83	0.005 **	0.07	−0.09, −0.02

*Note*. Number of subjects (N), mean (M), standard deviation (sd), Wilcoxon test (Z), level of significance (p), effect size (r) and confidence interval (CI). * <0.05, ** <0.01.

## Data Availability

The data that support the findings of this study are available on request from the corresponding author. The data are not publicly available due to privacy and ethical reasons.
